# Acute Effects of Dietary Carbohydrate Restriction on Glycemia, Lipemia and Appetite Regulating Hormones in Normal-Weight to Obese Subjects

**DOI:** 10.3390/nu10091285

**Published:** 2018-09-12

**Authors:** Amirsalar Samkani, Mads J. Skytte, Mads N. Thomsen, Arne Astrup, Carolyn F. Deacon, Jens J. Holst, Sten Madsbad, Jens F. Rehfeld, Thure Krarup, Steen B. Haugaard

**Affiliations:** 1 Department of Endocrinology, Copenhagen University Hospital, Bispebjerg, DK-2400 Copenhagen NV, Denmark; madsskytte@hotmail.com (M.J.S.); madsnorvinthomsen@gmail.com (M.N.T.); krarupthure@gmail.com (T.K.); d299057@dadlnet.dk (S.B.H.); 2 Department of Nutrition, Exercise and Sports, University of Copenhagen, DK-1017 Copenhagen K, Denmark; ast@nexs.ku.dk; 3 Endocrinology Research Section, Department of Biomedical Sciences, University of Copenhagen, DK-1017 Copenhagen K, Denmark; deacon@sund.ku.dk (C.F.D.); jjholst@sund.ku.dk (J.J.H.); 4 Section for Translational Physiology, NNF Center for Basic Metabolic Research, University of Copenhagen, DK-1017 Copenhagen K, Denmark; 5 Department of Endocrinology, Copenhagen University Hospital, Amager Hvidovre, DK-2650 Hvidovre, Denmark; Sten.Madsbad@regionh.dk; 6 Department of Clinical Biochemistry, Copenhagen University Hospital, Rigshospitalet, DK-2100 Copenhagen, Denmark; jens.f.rehfeld@regionh.dk; 7 Department of Internal Medicine, Copenhagen University Hospital, Amager Hvidovre, DK-2650 Hvidovre, Denmark

**Keywords:** carbohydrate reduction, postprandial glucose metabolism, second-meal effect

## Abstract

Postprandial responses to food are highly dependent on the macronutrient composition of the diet. We investigated the acute effects of transition from the recommended moderately high carbohydrate (HC) diet towards a carbohydrate-reduced high-protein (CRHP) diet on postprandial glycemia, insulinemia, lipemia, and appetite-regulating hormones in non-diabetic adults. Fourteen subjects, including five males (Mean ± SD: age 62 ± 6.5; BMI 32 ± 7.6 kg/m^2^; hemoglobin A_1c_ (HbA_1c_) 40 ± 3.0 mmol/mol; HOMA2-IR 2.1 ± 0.9) were included in this randomized, cross-over study. Iso-caloric diets were consumed for two consecutive days with a median wash-out period of 21 days (range 2–8 weeks) between diets (macronutrient energy composition: CRHP/HC; 31%/54% carbohydrate, 29%/16% protein, 40%/30% fat). Postprandial glucose, insulin secretion rate (ISR), triglycerides (TGs), non-esterified fatty acids (NEFAs), and satiety ratings were assessed after ingestion of breakfast (Br) and lunch (Lu), and gut hormones and glucagon were assessed after ingestion of Br. Compared with the HC diet, the CRHP diet reduced peak glucose concentrations (Br 11%, *p* = 0.024; Lu 11%, *p* < 0.001), glucose excursions (Br 80%, *p* = 0.20; Lu 85%, *p* < 0.001), and ISR (Br 31%; Lu 64%, both *p* < 0.001) whereas CRHP, as compared with HC, increased glucagon-like peptide-1 (Br 27%, *p* = 0.015) and glucagon values (Br 249%, *p* < 0.001). NEFA and TG levels increased in the CRHP diet as compared with the HC diet after Br, but no difference was found after Lu (NEFA Br 22%, *p* < 0.01; TG Br 42%, *p* = 0.012). Beta-cell glucose sensitivity, insulin clearance, cholecystokinin values, and subjective satiety ratings were unaffected. It is possible to achieve a reduction in postprandial glycemia and insulin without a deleterious effect on beta-cell glucose sensitivity by substituting part of dietary carbohydrate with iso-caloric protein and fat in subjects without type 2 diabetes mellitus (T2DM). The metabolic effects are more pronounced after the second meal.

## 1. Introduction

Obesity, as well as impaired glucose tolerance with a pre-diabetic hemoglobin A_1c_ (HbA_1c_), are associated with increasing risk of developing type 2 diabetes mellitus (T2DM) and coronary heart disease [1,2]. These conditions are often characterized by elevated circulating glucose and insulin concentrations [3,4]. Dietary interventions combined with increased physical activity remain the conventional first-line approaches to treatment of the pre-diabetic state [5,6]. Whereas the effects of weight loss and exercise develop gradually, a change in macronutrient composition might induce more immediate results as the postprandial response is affected acutely [7,8,9]. The macronutrient composition of the diet has been shown to play a significant role in satiety, gut hormone secretion, glucose metabolism, and insulin secretion in healthy adults and in subjects with type 2 diabetes mellitus (T2DM) [7,8,10,11,12]. Intake of large amounts of carbohydrates with high glycemic index have been associated with development of T2DM and coronary heart disease in healthy adults [4,13,14,15,16]. As hyperinsulinemia can induce insulin resistance and postprandial hyperglycemia contributes to elevated HbA_1c_, dietary carbohydrate reduction may be beneficial for subjects at risk for developing T2DM by reducing postprandial excursions of glucose and insulin [4,17,18,19,20]. When reducing dietary carbohydrates under eucaloric conditions, protein and fat must be added to the diet. High-fat diets have been thought to cause increases in blood lipids and to increase the risk of coronary heart disease, although this has been highly debated [21,22,23,24,25].

The aim of the present mechanistic study was to examine the acute effects on glucose and lipid metabolism of a carbohydrate-reduced high-protein (CRHP) diet compared with an energy-matched currently recommended high-carbohydrate (HC) diet [26,27] in obese and non-obese subjects with normal or pre-diabetic HbA_1c_ in subsequent meals. Breakfast and lunch meals were chosen to assess second-meal phenomenon, as the difference in duration from the last meal is the greatest for these two meals during the day. Moreover, incretin hormones, satiety, and gut hormones related to satiety were measured, as they play a role in postprandial satiety and glucose homeostasis [28,29].

## 2. Materials and Methods

### 2.1. Study Design

The study was designed as a, controlled, cross-over study with two arms where the subjects received the CRHP and the HC diets in randomized order. Each diet was provided for two consecutive days with a wash-out period of 2 to 8 weeks between interventions. Randomization was performed by a third-party study nurse by drawing blinded ballots. Participants were provided with breakfast and lunch to be ingested at the study site (Endocrine Research Unit at Copenhagen University Hospital at Bispebjerg, Copenhagen, Denmark), while dinner and pre- and post-dinner snacks were provided to be consumed at home. The breakfast and lunch meals also served as mixed meal tests (MMT). Diets were the same on day 1 and day 2. The evening prior to the intervention days, subjects were provided a standardized dinner to be ingested at home. Thirty percent of the subject’s calculated energy expenditure was ingested at breakfast, 30% at lunch and 30% at dinner. The remaining 10% percent was ingested as pre- and post-dinner snacks respectively. For the three days immediately before the interventions, subjects were asked to adjust their carbohydrate intake to 150–300 g daily and to refrain from strenuous physical activity and alcohol intake. On intervention days, no tea or coffee was allowed, and only sedentary activities were permitted. The methods employed for the present study were the same as employed and described in detail in an earlier study of subjects with type 2 diabetes [8]. The study was registered at clinicaltrials.gov (ID:NCT02472951) and was approved by The Danish National Committee on Health Research Ethics in accordance with the Helsinki II declaration. Before any study related procedures were initiated, written informed consent was obtained from all subjects. All subjects were non-smokers and all female participants were post-menopausal. All subjects were weight-stable prior to and throughout the study. Subjects with critical illness, renal or liver disease, steroid treatment, food allergy or intolerance, gut disease, or alcohol abuse were excluded.

### 2.2. Diet Compositions

The CRHP diet consisted of 31% energy from carbohydrates, 29% energy from protein, and 40% energy from fat, compared with 54% from carbohydrate, 16% from protein, and 30% from fat in the HC diet (Table A1). The diets were energy-matched and weighed out individually for each subject according to their estimated daily caloric expenditure, by trained kitchen personnel at Copenhagen University Hospital, Bispebjerg. Each subject’s daily energy requirement was assessed by calculation of daily resting energy expenditure (REE) based on a dual-energy x-ray absorptiometry scan (Lunar iDXA; GE Healthcare, Madison, WI, USA) [30]. As subjects were sedentary throughout the intervention days, REE was multiplied with a physical activity level of 1.4 to calculate daily total energy expenditure (TEE).

#### The Mixed Meal Tests

After a 10–12 h overnight fast, subjects reported to the Endocrine Research Unit and were placed in a reclining position after voiding the bladder and being weighed. A venous catheter was placed in an antecubital vein to draw blood samples at time points: −10, 0, 10, 20, 30, 45, 60, 90, 120, 150, 180, 210, 240, 270, 280, 290, 300, 315, 330, 360, 390, 420, and 450 min. Breakfast was ingested between times 0–30 min and lunch between times 270–300 min. Glucose, insulin, C-peptides, non-esterified fatty acids (NEFAs), and triglycerides (TGs) were measured at all time points, while glucagon, glucagon-like peptide-1 (GLP-1), and cholecystokinin (CCK) were measured at the following time points: 0, 30, 60, 90, 120, 150, 180, and 240 min. Satiety was assessed at 0, 30, 60, 120, 180, 240, 270, 300, 300, 330, 390, and 450 min and expressed as a composite satiety score (CSS) based on 100-mm visual analogue scales (VAS) with four questions to integrate appetite sensations into one mean index (range 0–100 mm) [7,8,31,32].

### 2.3. Analytical Procedures

Of each blood sample drawn, the first 2 mL were discarded. Serum was obtained by collecting blood in clot activator tubes left for 30 min at room temperature before centrifugation and plasma was prepared from blood sampled in pre-chilled EDTA tubes, which were centrifuged immediately at 4 °C. Insulin, C-peptides, NEFA, and TG values were analyzed in serum, while glucose, GLP-1, glucagon, and CCK values were analyzed in plasma. Analytical methods have been described in detail previously [8]. In short, glucose was analyzed with YSI 2300 STAT plus (Yellow Spring Instruments). Insulin and C-peptides were analyzed with the IMMULITE 200 Immunoassay System (Siemens Healthcare). NEFA values were analyzed with the ACS-ACOD Method [33,34] by using a commercially available reagent (Wako, NEFA-HR (2), Wako Chemicals GmbH, Neuss, Germany). TG values were analyzed using an enzymatic colorimetric analysis on the Cobas 8000 modular analyzer (Roche Diagnostics, Indianapolis, IN, USA), standardized against isotope-dilution mass spectrometry [35]. Plasma samples were extracted with ethanol (70% *v*/*v*), for analysis of GLP-1 and glucagon. Antiserum code no. 89390 was used to measure GLP-1 [36], antiserum code no. 4305 was used to measure glucagon [37] and antiserum code no. 92128 to measure CCK [38].

### 2.4. Statistical Analysis

Means of both days on each diet were calculated. For all fourteen subjects, results, graphs and statistical analyses are presented as means of the two consecutive days on each diet. Fasting concentrations were measured at time 0 min or as a mean of time −10 and 0 min when applicable (glucose, insulin, C-peptides, NEFAs, and TGs). The trapezoidal rule was used to calculate area under curve (AUC) and by subtracting area below individual fasting concentrations from AUC, netAUC was calculated. Peak concentrations were identified for each individual for all variables (except for NEFA where the nadir concentration was identified). A software program, ISEC (Insulin SECretion), was used to calculate prehepatic insulin secretion rates (ISR) by deconvolution of C-peptide concentrations [39,40]. As a measure of initial β-cell glucose sensitivity (β-GS), change in ISR per unit change in glucose concentration (ΔISR/Δglucose) from baseline to peak glucose concentration was calculated for each subject during the breakfast and lunch meals, respectively. Insulin clearance during meals was calculated as an index (AUC_insulin_/AUC_ISR_).

If data followed a Gaussian distribution, as determined by the Shapiro–Wilk normality test, results are presented as means with their standard errors (SEM) and if not, results are presented as medians and interquartile range (IQR). Student’s paired *t*-test was used to calculate simple differences (AUC, peaks etc.) between diets, and Wilcoxon-matched pairs signed rank test was used if a Gaussian distribution was not found. After subtracting baseline values, two-way repeated-measures ANOVA with time and treatment as repeated measures and subjects as fixed effects were used to test for postprandial differences at different time points. To adjust for multiple comparisons at each time point post hoc, Bonferroni’s multiple comparison adjustment was used. Significance level was set to *p* = 0.05. Graphpad Prism for Windows (version 7.02, 13 September 2016; Graphpad software, La Jolla, CA, USA) was used for statistical analyses and graphical presentations.

## 3. Results

### 3.1. Subjects

Fourteen subjects ranging from normal weight-to-obese (BMI range: 22.7–49.3 kg/m^2^) without T2DM (HbA_1c_ range: 35–45) were included in the study (Table 1).

No differences were found in fasting concentrations on the different treatment days on any of the measured variables.

### 3.2. Glucose

**Figure 1:**
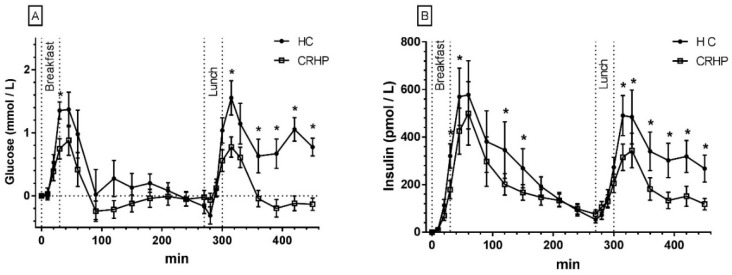
Mean ± SEM 7.5-h concentrations of glucose (**A**) and insulin (**B**) in 14 non-diabetic subjects after intake of a CRHP or HC breakfast and lunch, respectively (mean of two consecutive days on each diet). * Significant difference (*p* < 0.05) between the HC and CRHP diet. HC: high carbohydrate; CRHP: carbohydrate-reduced high-protein.

An interaction between time and treatment was found in the 7.5-h repeated measures analysis of glucose (*p* < 0.001). The CRHP diet reduced peak glucose concentrations by 11% (0.8 ± 0.3 mmol/L, *p* = 0.024) after ingestion of breakfast and by 11% (0.9 ± 0.2 mmol/L, *p* < 0.001) after ingestion of lunch, respectively, compared with the HC diet. On both diets, peak glucose concentrations were reached 45 min after ingestion of breakfast and lunch, respectively (Figure 1A).

A non-significant reduction in glucose breakfast AUC of 6% and netAUC of 80% was found on the CRHP compared with HC diet (0.3 ± 0.2 mmol/L × 270 min, *p* = 0.13, and 0.3 ± 0.2 mmol/L × 270 min, *p* = 0.20, respectively). Compared with intake of the HC diet, the CRHP diet reduced lunch glucose AUC by 12% (0.8 ± 0.1 mmol/L × 180 min, *p* < 0.001) and netAUC by 85% (0.8 ± 0.2 mmol/L × 180 min, *p* < 0.001).

### 3.3. Insulin

An interaction between time and treatment was found in the 7.5-h repeated measures analysis of insulin (*p* < 0.001). The CRHP diet reduced median peak insulin concentration by 32% (201 (IQR 12–331) pmol/L, *p* = 0.042) after ingestion of breakfast and by 33% (154 (IQR 62–243) pmol/L, *p* = 0.005) after ingestion of lunch, respectively, compared with the HC diet. On both diets, peak insulin concentration was reached 60 min after ingestion of breakfast and after 60 min on the CRHP diet compared with 45 min on the HC diet after ingestion lunch (Figure 1B).

Compared with the HC diet, ingestion of the CRHP diet reduced breakfast median insulin netAUC by 29% (51 (IQR 21–104) pmol/L × 270 min, *p* = 0.049) and lunch median insulin netAUC by 63% (123 (IQR 69-188) pmol/L × 180 min, *p* < 0.001), respectively.

### 3.4. C-Peptides

**Figure 2:**
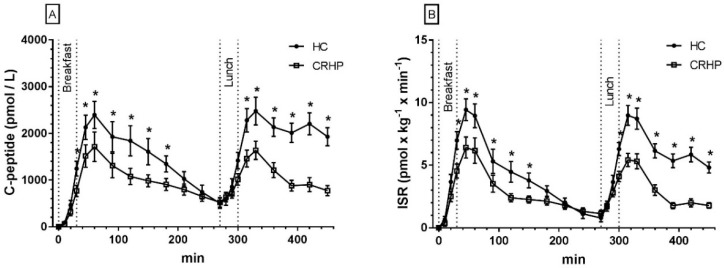
Mean ± SEM 7.5-h concentrations of C-peptides (**A**) and ISR (**B**) in 14 non-diabetic subjects after intake of a CRHP or HC breakfast and lunch, respectively (mean of two consecutive days on each diet). * Significant difference (*p* < 0.05) between the HC and CRHP diet. HC: high carbohydrate; CRHP: carbohydrate-reduced high-protein; ISR: insulin secretion rate.

An interaction between time and treatment was found in the 7.5-h repeated measures analysis of C-peptides (*p* < 0.001). The CRHP diet reduced peak C-peptide concentrations by 23% (802 ± 183 pmol/L, *p* < 0.001) after ingestion of breakfast and by 29% (1026 ± 103 pmol/L, *p* < 0.001) after ingestion of lunch, respectively, compared with the HC diet. Peak C-peptide concentration was reached 60 min after ingestion of both breakfast and lunch, respectively, on both diets (Figure 2A).

Compared with intake of the HC diet, the CRHP diet reduced breakfast C-peptide netAUC by 31% (432 ± 97 pmol/L × 270 min) and lunch C-peptide netAUC by 63% (890 ± 139 pmol/L × 180 min) (both *p* < 0.001).

### 3.5. Insulin Secretion Rate

As ISR was calculated by deconvolution of C-peptides, an interaction between time and treatment was found in the 7.5-h repeated measures analysis of ISR (*p* < 0.001). The CRHP diet, as compared with the HC diet, reduced peak ISR by 27% (3.5 ± 0.7 pmol/kg per min) after ingestion of breakfast and by 31% (3.6 ± 0.4 pmol/kg per min) after ingestion of lunch (both *p* < 0.001). Peak ISR was reached 60 min after ingestion of both breakfast and lunch on both diets (Figure 2B).

Intake of the CRHP diet, as compared with HC diet, reduced breakfast ISR netAUC by 31% (1.3 ± 0.2 pmol/L × 270 min, *p* < 0.001) and lunch ISR netAUC by 64% (3.3 ± 0.5 mmol/L × 180 min, *p* < 0.001).

### 3.6. β-Cell Glucose Sensitivity and Insulin Clearance

No difference in initial β-GS was found after ingestion of CRHP compared with HC breakfast (5.7 ± 1.0 vs. 5.1 ± 0.6 pmol/kg per min per mM, *p* = 0.60) or lunch (5.3 ± 0.8 vs. 4.3 ± 0.6 pmol/kg per min per mM, *p* = 0.34), respectively.

No difference in insulin clearance index was found after ingestion of the CRHP compared with HC breakfast (44 (IQR 28–67) vs. 42 (IQR 32–64) kg/l × min, *p* = 0.43) or lunch (40 (IQR 30–62) vs. 40 (IQR 30–54) kg/L × min, *p* =0.33), respectively.

### 3.7. Non-Esterified Fatty Acids

**Figure 3:**
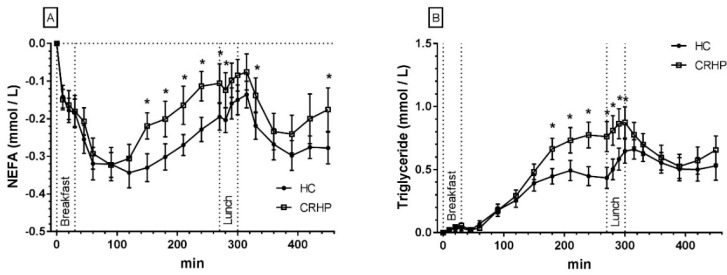
Mean ± SEM 7.5-h concentrations of NEFAs (**A**) and triglycerides (**B**) in 14 non-diabetic subjects after intake of a CRHP or HC breakfast and lunch, respectively (mean of two consecutive days on each diet). * Significant difference (*p* < 0.05) between the HC and CRHP diet. HC: high carbohydrate; CRHP: carbohydrate-reduced high-protein; NEFA: non-esterified fatty acid.

An interaction between time and treatment was found in the 7.5-h repeated measures analysis of NEFA (*p* < 0.001). The CRHP diet increased nadir NEFA concentration by 20% (0.30 ± 0.02 vs. 0.25 ± 0.02 mmol/L, *p* = 0.027) after ingestion of breakfast and by 29% (0.37 ± 0.03 vs. 0.29 ± 0.02 mmol/L, *p* = 0.001) after ingestion of lunch, respectively, compared with the HC diet. Nadir NEFA concentration was reached after 90 min on the CRHP diet compared with after 120 min on the HC diet after ingestion of breakfast and 120 min after ingestion of lunch on both diets, respectively (Figure 3A).

Compared with intake of the HC diet, the CRHP diet increased breakfast median NEFA netAUC by 22% (0.06 (IQR 0.01–0.08) mmol/L × 270 min, *p* = 0.005). No difference was found in lunch NEFA netAUC between diets.

### 3.8. Triglycerides

An interaction between time and treatment was found in the 7.5-h repeated measures analysis of TG (*p* < 0.001). No difference in peak TG concentrations was found between diets after ingestion of either breakfast or lunch. Peak TG concentration was reached after 240 min on the CRHP diet compared with after 210 min on the HC diet after ingestion of breakfast and after 30 min on the CRHP diet compared with 45 min on the HC diet after ingestion of lunch, respectively (Figure 3B).

Compared with intake of the HC diet, the CRHP diet increased breakfast TG netAUC by 42% (0.12 ± 0.04 mmol/L × 270 min, *p* = 0.012). No difference was found in lunch TG AUC between diets.

### 3.9. Glucagon-Like Peptide-1

**Figure 4:**
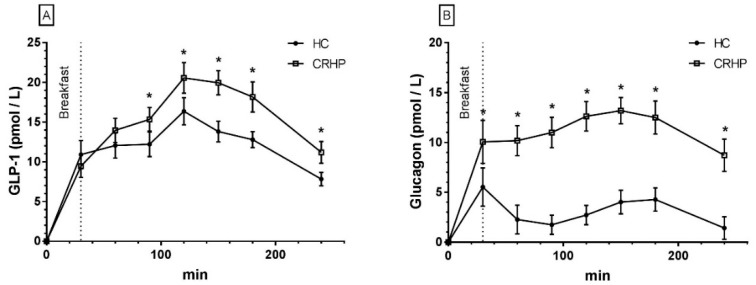
Mean ± SEM 4-h concentrations of GLP-1 (**A**) and glucagon (**B**) in 14 non-diabetic subjects after intake of a CRHP or HC breakfast, respectively (mean of 2 consecutive days on each diet). * Significant difference (*p* < 0.05) between the HC and CRHP diet. HC: high carbohydrate; CRHP: carbohydrate-reduced high-protein; GLP-1: glucagon-like peptide-1.

An interaction between time and treatment was found in the 4-h repeated measures analysis of GLP-1 (*p* < 0.001). GLP-1 concentrations were significantly higher 90–240 min after ingestion of the CRHP compared with the HC breakfast. Peak concentration was reached 120 min after ingestion of both diets. A 17% (4.2 ± 1.9 pmol/L, *p* = 0.045) higher peak was found in the plasma concentration of GLP-1 after ingestion of the CRHP compared with HC breakfast (Figure 4A). Likewise, the GLP-1 netAUC value was 27% (3.2 ± 1.1 pmol/L × 240 min, *p* = 0.015) higher after ingestion of the CRHP compared with the HC breakfast.

### 3.10. Glucagon

A time-treatment interaction was found in the 4-h repeated measures analysis of glucagon (*p* < 0.001). Compared with the HC diet, ingestion of the CRHP breakfast significantly increased glucagon concentration at all time points, increased peak concentration by 39% (7.2 ± 1.2 pmol/L, *p* < 0.001) and netAUC by 249% (7.5 ± 1.2 pmol/L, *p* < 0.001) (Figure 4B).

### 3.11. Cholecystokinin

**Figure 5:**
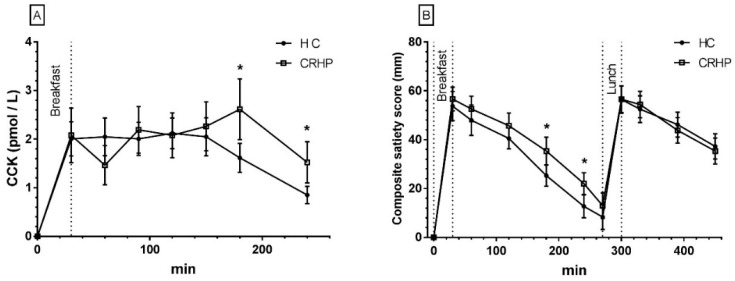
Mean ± SEM 4-h concentrations of CCK (**A**) in 14 non-diabetic subjects after intake of a CRHP or HC breakfast and 7.5-h composite satiety score on a 100-mm VAS scale (**B**) after intake of a CRHP or HC breakfast and lunch, respectively (mean of two consecutive days on each diet). * Significant difference (*p* < 0.05) between HC and CRHP diet. HC: high carbohydrate; CRHP: carbohydrate-reduced high-protein; CCK: cholecystokinin; VAS: visual analogue scale.

A time-treatment interaction was found in the 4-h repeated measures analysis of CCK (*p* < 0.01). Compared with the HC diet, ingestion of the CRHP breakfast significantly increased CCK concentration at individual time points 180 and 240 min, although no significant differences were found in peak concentration or netAUC between diets (Figure 5A).

### 3.12. Composite Satiety Score

A trend for interaction was found between time and treatment in the 7.5-h repeated measures analysis of CSS (*p* = 0.057). At individual times of measurement, satiety was significantly higher 180 and 240 min after ingestion of the CRHP compared with HC breakfast. No difference in peak satiety score was found between diets. On both diets, peak satiety was reached 30 min after ingestion of breakfast and lunch, respectively (Figure 5B). No difference between diets was found in CSS breakfast or lunch netAUC.

### 3.13. Explorative Analyses

No correlation was found between HOMA2-IR, BMI, HbA_1c,_ or fasting glucose and effect size of reduction in postprandial glucose excursion or insulin secretion (Spearman *p* > 0.2 for all). No difference was found in effect size of reduction in postprandial glucose excursion or insulin secretion after stratification of subjects in two equal size groups based on HbA_1c_ (35–40 and 41–45), BMI (<30 and >30), and fasting glucose (<6 and >6) (all *p* > 0.05).

## 4. Discussion

The major new finding in this study in obese and non-obese subjects with prediabetes or normal glucose tolerance was a highly significant and clinically relevant acute reduction in postprandial insulin and C-peptide responses on a diet reduced in carbohydrate and increased in protein and fat. This was found both after ingestion of breakfast and lunch without altering β-cell glucose sensitivity and insulin clearance. Furthermore, the postprandial glucose excursion was reduced following lunch and glucose peak concentrations were reduced following both breakfast and lunch meals. This is important as postprandial glucose excursions contribute relatively more to HbA_1c_ in subjects with lower fasting glucose concentrations [17,18] and may suggest that remission of prediabetes can be achieved with a carbohydrate-reduced high-protein diet in obese adults [19].

We have previously shown that the hallmark of early T2DM, i.e., elevated glucose and insulin excursions following meals, can be ameliorated by a reduction in dietary carbohydrate content with iso-caloric replacement of fat and protein [8]. The findings in the present study show that similar effects may also be found in subjects without T2DM. Reduction of postprandial hyperinsulinemia and hyperglycemia may be an effective strategy to prevent development of insulin resistance and obesity in subjects at risk, as both have been associated with obesity and development of diabetes [16,41,42,43,44,45]. Although more research is needed to establish a causal relation, hyperinsulinemia has been associated with a plethora of pathological conditions, e.g., hypertension, malignancy, stroke and coronary heart disease, implying a possible benefit of less insulinogenic diets for subjects at risk for these conditions [42,46,47,48,49,50]. Furthermore, it is important to note that no acute adverse effect was found in β-cell glucose sensitivity on the CRHP diet despite a reduced insulin excursion and a reduced suppression of circulating NEFA concentrations after breakfast.

NEFA levels were less suppressed after intake of the CRHP compared with HC at breakfast, while both diets elicited a similar postprandial decrease of NEFA after ingestion of lunch. The difference in postprandial NEFA excursions following breakfast can be partly explained by the suppression of lipolysis and increased re-esterification of NEFA by insulin [51,52], while the excursion following lunch must be interpreted together with the excursion in TG because insulin also increases lipoprotein lipase (LPL) activity after a delay, causing some NEFAs from TG-rich lipoproteins to ‘spill-over’ back to the circulation at the site of action, i.e., the luminal surface of endothelial cells in capillaries [53,54,55,56,57]. Much to our surprise, the same trend was found in the postprandial triglyceride excursions, i.e., the postprandial TG excursion was increased by the CRHP as compared with the HC meal at breakfast but not lunch. Previous studies suggested this phenomenon to be caused by an increased postprandial insulin response to the HC diet, resulting in increased hepatic production rate of very low-density lipoprotein-TGs (VLDL-TGs). An increase in VLDL-TGs in turn reduces clearance of chylomicron-TG from the second meal, as both are cleared by LPL in a competitive manner [58,59,60]. The present study underscores the importance of interpreting these two important energy substrates in man in conjunction and during several meals to achieve a sufficient duration to assess the underlying hormonal regulation. Accordingly, a single meal test may be misleading and confound interpretation of the effect of the dietary interventions evaluated. This seemingly paradoxical pattern in postprandial TG excursions on sequential meals may explain the reduced fasting concentrations of TG on low carbohydrate diets in long term trials found in a recent review [61].

As expected, glucagon responses were increased by the CRHP compared with HC diet due to the higher content of protein, as seen in previous studies [7,8,62]. GLP-1 responses also increased, reflecting the fact that both protein and fat are potent stimuli for GLP-1 secretion. In our previous study in subjects with T2DM, the CRHP diet, as compared with HC diet, did not increase GLP-1 levels, leading us to speculate if the GLP-1 response to protein and fat ingestion might be blunted in subjects with T2DM [8]. As both glucagon and GLP-1 have been associated with satiation [7,63,64], these responses could perhaps explain the higher satiety scores found at 180 and 240 min after ingestion of breakfast on the CRHP diet in the present study. Furthermore, the increase in GLP-1 secretion may, in part, explain the maintained β-cell glucose sensitivity despite the reduced glucose excursion after CRHP diet ingestion. The CCK response was increased 180 and 240 min after ingestion of the CRHP compared with HC breakfast due to increased fat content [65] in the CRHP (40E%) compared with HC (30E%) diet and coincided with the increase in satiety scores at these time points. Interestingly, we found, in our previous study, that CCK was increased to a larger extent by a CRHP compared with HC diet in subjects with T2DM concurrently with increased satiety. The putative mechanism might be an exacerbated response of CCK and thus satiety to increased fat in subjects with T2DM [9].

A limitation of the present study is the broad metabolic range of subjects regarding glucose metabolism, which could confound the findings and interpretation of results, but no correlation was found between degree of impaired glucose tolerance (e.g., HOMA2-IR, HbA_1c_, or fasting glucose) and effect size of reduction in postprandial glucose excursion or insulin secretion, though.A strength of the present study is the measurement of postprandial responses to two subsequent meals, as the response to a second meal can differ from the response to the first meal, as discussed above.

## 5. Conclusions

Our group has previously found reduced hyperglycemia and hyperinsulinemia and increased satiety on a CRHP diet in subjects with T2DM. The present study suggests a similar promising effect in non-diabetic subjects. Future studies are needed to evaluate the complete diurnal postprandial effects of a shift in macronutrient composition in subjects with and without T2DM and long-term studies are needed to evaluate whether these effects are sustained or even amplified over time. As adherence is a major issue in dietary trials, long-term studies should emphasize the importance of adherence, for instance through diet provision, in evaluations of the true potential for carbohydrate-reduced diets to prevent or treat T2DM.

## Figures and Tables

**Table 1:** Baseline characteristics of participants.

Subject (no.)	Age (years)	Gender (M/F ^1^)	Weight (kg)	BMI ^2^ (kg/m^2^)	HbA_1c_^3^ (mmol/mol)	Fasting PG ^4^ (mmol/L)	HOMA2-IR ^5^	TEE ^6^ (MJ/day)
1	45	M	138	43.4	39	6.2	3.2	13.6
2	70	F	127	49.3	41	6.6	3.4	11.0
3	70	F	108	38	45	6.3	3.6	10.1
4	63	F	109	34.1	42	6.3	2.1	10.0
5	65	F	88	34.7	39	6.2	2.4	8.5
6	59	F	77	30.8	42	5.8	1.6	8.3
7	56	M	121	34.9	42	5.4	3	12.2
8	59	M	70	22.7	35	5.2	1	8.8
9	63	F	80	28.3	37	5.0	1.8	8.3
10	64	F	67	23.8	35	5.0	1.2	7.8
11	67	M	103	28.4	38	6.2	2.1	11.0
12	56	M	75	25.2	43	6.1	1.1	9.7
13	63	F	90	27.9	40	5.8	1.7	9.3
14	64	F	78	27.6	42	5.4	1	8.3
Mean	61.7	5 M/9 F	95	32.1	40	5.8	2.1	9.8
Range	45–70	5 M/9 F	78–138	22.7–49.3	35–45	5.0–6.6	1–3.6	8.3–13.6

^1^ Male/female; ^2^ body mass index; ^3^ hemoglobin A_1c_; ^4^ Plasma glucose; ^5^ HOMA2 Calculator Version 2.2.3 (University of Oxford, Oxford, UK); ^6^ Total energy expenditure.

## Appendix A

**Table A1:** Macronutrient composition and ingredients of the test meals standardized at 10 MJ/day.

	CRHP ^1^ Diet	HC ^2^ Diet
Breakfast		
Energy (kJ)	3000	3000
Carbohydrate (E%)	31	54
Protein (E%)	29	16
Fat (E%)	40	30
Fiber (g/MJ)	2.5	4
Ingredients (g)		
Egg	192.3	39.7
Olive oil	7.5	-
Bread	37.4	69.4
Rye flour yoghurt topping	21.4	49.6
Tomato	85.5	-
Cheese	16.0	19.8
Ham	26.7	-
Skyr (icelandic yoghurt) with vanilla	160.3	-
Strawberry jam	-	19.7
Apple	-	49.6
Almond	-	11.9
Milk, acidophilus cultured	-	198.3
**Lunch**		
Energy (kJ)	3000	3000
Carbohydrate (E%)	31	54
Protein (E%)	29	16
Fat (E%)	40	30
Fiber (g/MJ)	3.5	2.7
Ingredients (g)		
Chicken	137.3	38.1
Olive oil	14.7	-
Tomato	147.2	142.8
Spring onion	9.8	19.0
Bell pepper	29.4	47.6
Bread	24.5	47.6
Milk	245.3	142.8
Feta cheese	29.4	-
Chick peas	39.2	-
Pasta	-	66.6
Pesto	-	33.3
Butter	-	9.5

^1^ CRHP, carbohydrate-reduced high-protein; ^2^ HC, high carbohydrate.

## References

[1] Hu F.B., Manson J.E., Stampfer M.J., Colditz G., Liu S., Solomon C.G., Willett W.C. (2001). Diet, lifestyle, and the risk of type 2 diabetes mellitus in women. N. Engl. J. Med..

[2] Huang Y., Cai X., Mai W., Li M., Hu Y. (2016). Association between prediabetes and risk of cardiovascular disease and all cause mortality: Systematic review and meta-analysis. BMJ.

[3] Stratton I.M., Adler A.I., Neil H.A., Matthews D.R., Manley S.E., Cull C.A., Hadden D., Turner R.C., Holman R.R. (2000). Association of glycaemia with macrovascular and microvascular complications of type 2 diabetes (UKPDS 35): Prospective observational study. BMJ.

[4] Ludwig D.S., Hu F.B., Tappy L., Brand-Miller J. (2018). Dietary carbohydrates: Role of quality and quantity in chronic disease. BMJ.

[5] American Diabetes Association (2018). 4. Lifestyle management: Standards of medical care in diabetes-2018. Diabetes Care.

[6] American Diabetes Association (2018). 5. Prevention or delay of type 2 diabetes: Standards of medical care in diabetes-2018. Diabetes Care.

[7] Belza A., Ritz C., Sorensen M.Q., Holst J.J., Rehfeld J.F., Astrup A. (2013). Contribution of gastroenteropancreatic appetite hormones to protein-induced satiety. Am. J. Clin. Nutr..

[8] Samkani A., Skytte M.J., Kandel D., Kjaer S., Astrup A., Deacon C.F., Holst J.J., Madsbad S., Rehfeld J.F., Haugaard S.B. (2018). A carbohydrate-reduced high-protein diet acutely decreases postprandial and diurnal glucose excursions in type 2 diabetes patients. Br. J. Nutr..

[9] Steinert R.E., Feinle-Bisset C., Asarian L., Horowitz M., Beglinger C., Geary N. (2017). Ghrelin, CCK, GLP-1, and PYY (3–36): Secretory controls and physiological roles in eating and glycemia in health, obesity, and after rygb. Physiol Rev..

[10] Pearce K.L., Noakes M., Keogh J., Clifton P.M. (2008). Effect of carbohydrate distribution on postprandial glucose peaks with the use of continuous glucose monitoring in type 2 diabetes. Am. J. Clin. Nutr..

[11] Nuttall F.Q., Almokayyad R.M., Gannon M.C. (2015). Comparison of a carbohydrate-free diet vs. Fasting on plasma glucose, insulin and glucagon in type 2 diabetes. Metabolism.

[12] Snorgaard O., Poulsen G.M., Andersen H.K., Astrup A. (2017). Systematic review and meta-analysis of dietary carbohydrate restriction in patients with type 2 diabetes. BMJ Open Diabetes Res. Care.

[13] Salmeron J., Ascherio A., Rimm E.B., Colditz G.A., Spiegelman D., Jenkins D.J., Stampfer M.J., Wing A.L., Willett W.C. (1997). Dietary fiber, glycemic load, and risk of niddm in men. Diabetes Care.

[14] Salmeron J., Manson J.E., Stampfer M.J., Colditz G.A., Wing A.L., Willett W.C. (1997). Dietary fiber, glycemic load, and risk of non-insulin-dependent diabetes mellitus in women. JAMA.

[15] Liu S., Willett W.C., Stampfer M.J., Hu F.B., Franz M., Sampson L., Hennekens C.H., Manson J.E. (2000). A prospective study of dietary glycemic load, carbohydrate intake, and risk of coronary heart disease in us women. Am. J. Clin. Nutr..

[16] Willett W., Manson J., Liu S. (2002). Glycemic index, glycemic load, and risk of type 2 diabetes. Am. J. Clin. Nutr..

[17] Monnier L., Lapinski H., Colette C. (2003). Contributions of fasting and postprandial plasma glucose increments to the overall diurnal hyperglycemia of type 2 diabetic patients: Variations with increasing levels of HBA_1c_. Diabetes Care.

[18] Monnier L., Colette C., Owens D. (2011). Postprandial and basal glucose in type 2 diabetes: Assessment and respective impacts. Diabetes Technol. Ther..

[19] Stentz F.B., Brewer A., Wan J., Garber C., Daniels B., Sands C., Kitabchi A.E. (2016). Remission of pre-diabetes to normal glucose tolerance in obese adults with high protein versus high carbohydrate diet: Randomized control trial. BMJ Open Diabetes Res. Care.

[20] Feinman R.D., Pogozelski W.K., Astrup A., Bernstein R.K., Fine E.J., Westman E.C., Accurso A., Frassetto L., Gower B.A., McFarlane S.I. (2015). Dietary carbohydrate restriction as the first approach in diabetes management: Critical review and evidence base. Nutrition.

[21] Parillo M., Rivellese A.A., Ciardullo A.V., Capaldo B., Giacco A., Genovese S., Riccardi G. (1992). A high-monounsaturated-fat/low-carbohydrate diet improves peripheral insulin sensitivity in non-insulin-dependent diabetic patients. Metabolism.

[22] Shai I., Schwarzfuchs D., Henkin Y., Shahar D.R., Witkow S., Greenberg I., Golan R., Fraser D., Bolotin A., Vardi H. (2008). Weight loss with a low-carbohydrate, mediterranean, or low-fat diet. N. Engl. J. Med..

[23] Estruch R., Ros E., Salas-Salvado J., Covas M.I., Corella D., Aros F., Gomez-Gracia E., Ruiz-Gutierrez V., Fiol M., Lapetra J. (2018). Primary prevention of cardiovascular disease with a mediterranean diet supplemented with extra-virgin olive oil or nuts. N. Engl. J. Med..

[24] Jeppesen J., Schaaf P., Jones C., Zhou M.Y., Chen Y.D., Reaven G.M. (1997). Effects of low-fat, high-carbohydrate diets on risk factors for ischemic heart disease in postmenopausal women. Am. J. Clin. Nutr..

[25] Harcombe Z., Baker J.S., DiNicolantonio J.J., Grace F., Davies B. (2016). Evidence from randomized controlled trials does not support current dietary fat guidelines: A systematic review and meta-analysis. Open Heart.

[26] Fogelholm M. (2013). New nordic nutrition recommendations are here. Food Nutr. Res..

[27] Mann J.I., De Leeuw I., Hermansen K., Karamanos B., Karlstrom B., Katsilambros N., Riccardi G., Rivellese A.A., Rizkalla S., Slama G. (2004). Evidence-based nutritional approaches to the treatment and prevention of diabetes mellitus. Nutr. Metab. Cardiovasc. Dis..

[28] Holst J.J., Knop F.K., Vilsboll T., Krarup T., Madsbad S. (2011). Loss of incretin effect is a specific, important, and early characteristic of type 2 diabetes. Diabetes Care.

[29] Madsbad S. (2014). The role of glucagon-like peptide-1 impairment in obesity and potential therapeutic implications. Diabetes Obes. Metab..

[30] Nielsen S., Hensrud D.D., Romanski S., Levine J.A., Burguera B., Jensen M.D. (2000). Body composition and resting energy expenditure in humans: Role of fat, fat-free mass and extracellular fluid. Int. J. Obes. Relat. Metab. Disord..

[31] Gibbons C., Finlayson G., Dalton M., Caudwell P., Blundell J.E. (2014). Metabolic phenotyping guidelines: Studying eating behavior in humans. J. Endocrinol..

[32] Chaput J.-P., Gilbert J.-A., Gregersen N.T., Pedersen S.D., Sjodin A.M. (2010). Comparison of 150-mm versus 100-mm visual analogue scales in free living adult subjects. Appetite.

[33] Rogiers V. (1978). Stability of the long chain non-esterified fatty acid pattern in plasma and blood during different storage conditions. Clin. Chim. Acta.

[34] Krebs M., Stingl H., Nowotny P., Weghuber D., Bischof M., Waldhausl W., Roden M. (2000). Prevention of in vitro lipolysis by tetrahydrolipstatin. Clin. Chem..

[35] Owen W.E., Thatcher M.L., Crabtree K.J., Greer R.W., Strathmann F.G., Straseski J.A., Genzen J.R. (2015). Body fluid matrix evaluation on a roche cobas 8000 system. Clin. Biochem..

[36] Orskov C., Rabenhoj L., Wettergren A., Kofod H., Holst J.J. (1994). Tissue and plasma concentrations of amidated and glycine-extended glucagon-like peptide I in humans. Diabetes.

[37] Lund A., Bagger J.I., Wewer Albrechtsen N.J., Christensen M., Grondahl M., Hartmann B., Mathiesen E.R., Hansen C.P., Storkholm J.H., van Hall G. (2016). Evidence of extrapancreatic glucagon secretion in man. Diabetes.

[38] Rehfeld J.F. (1998). Accurate measurement of cholecystokinin in plasma. Clin. Chem..

[39] Van Cauter E., Mestrez F., Sturis J., Polonsky K.S. (1992). Estimation of insulin secretion rates from C-peptide levels. Comparison of individual and standard kinetic parameters for C-peptide clearance. Diabetes.

[40] Hovorka R., Soons P.A., Young M.A. (1996). ISEC: A program to calculate insulin secretion. Comput. Methods Progr. Biomed..

[41] Koopmans S.J., Ohman L., Haywood J.R., Mandarino L.J., DeFronzo R.A. (1997). Seven days of euglycemic hyperinsulinemia induces insulin resistance for glucose metabolism but not hypertension, elevated catecholamine levels, or increased sodium retention in conscious normal rats. Diabetes.

[42] Modan M., Halkin H., Almog S., Lusky A., Eshkol A., Shefi M., Shitrit A., Fuchs Z. (1985). Hyperinsulinemia. A link between hypertension obesity and glucose intolerance. J. Clin. Investig..

[43] Mehran A.E., Templeman N.M., Brigidi G.S., Lim G.E., Chu K.Y., Hu X., Botezelli J.D., Asadi A., Hoffman B.G., Kieffer T.J. (2012). Hyperinsulinemia drives diet-induced obesity independently of brain insulin production. Cell Metab..

[44] Kaiser N., Leibowitz G., Nesher R. (2003). Glucotoxicity and beta-cell failure in type 2 diabetes mellitus. J. Pediatr. Endocrinol. Metab..

[45] Del Prato S., Leonetti F., Simonson D.C., Sheehan P., Matsuda M., DeFronzo R.A. (1994). Effect of sustained physiologic hyperinsulinaemia and hyperglycaemia on insulin secretion and insulin sensitivity in man. Diabetologia.

[46] Yoon Y.S., Keum N., Zhang X., Cho E., Giovannucci E.L. (2015). Hyperinsulinemia, insulin resistance and colorectal adenomas: A meta-analysis. Metabolism.

[47] Salonen J.T., Lakka T.A., Lakka H.M., Valkonen V.P., Everson S.A., Kaplan G.A. (1998). Hyperinsulinemia is associated with the incidence of hypertension and dyslipidemia in middle-aged men. Diabetes.

[48] Tsujimoto T., Kajio H., Sugiyama T. (2017). Association between hyperinsulinemia and increased risk of cancer death in nonobese and obese people: A population-based observational study. Int. J. Cancer.

[49] Pyorala M., Miettinen H., Laakso M., Pyorala K. (1998). Hyperinsulinemia and the risk of stroke in healthy middle-aged men: The 22-year follow-up results of the helsinki policemen study. Stroke.

[50] Pyorala M., Miettinen H., Laakso M., Pyorala K. (1998). Hyperinsulinemia predicts coronary heart disease risk in healthy middle-aged men: The 22-year follow-up results of the helsinki policemen study. Circulation.

[51] Boden G., Chen X., Desantis R.A., Kendrick Z. (1993). Effects of insulin on fatty acid reesterification in healthy subjects. Diabetes.

[52] Campbell P.J., Carlson M.G., Hill J.O., Nurjhan N. (1992). Regulation of free fatty acid metabolism by insulin in humans: Role of lipolysis and reesterification. Am. J. Physiol..

[53] Sadur C.N., Eckel R.H. (1982). Insulin stimulation of adipose tissue lipoprotein lipase. Use of the euglycemic clamp technique. J. Clin. Investig..

[54] Evans K., Burdge G.C., Wootton S.A., Clark M.L., Frayn K.N. (2002). Regulation of dietary fatty acid entrapment in subcutaneous adipose tissue and skeletal muscle. Diabetes.

[55] Pollare T., Vessby B., Lithell H. (1991). Lipoprotein lipase activity in skeletal muscle is related to insulin sensitivity. Arterioscler. Thromb..

[56] Ruge T., Hodson L., Cheeseman J., Dennis A.L., Fielding B.A., Humphreys S.M., Frayn K.N., Karpe F. (2009). Fasted to fed trafficking of fatty acids in human adipose tissue reveals a novel regulatory step for enhanced fat storage. J. Clin. Endocrinol. Metab..

[57] Miles J.M., Nelson R.H. (2007). Contribution of triglyceride-rich lipoproteins to plasma free fatty acids. Horm. Metab. Res..

[58] Chen Y.D., Coulston A.M., Zhou M.Y., Hollenbeck C.B., Reaven G.M. (1995). Why do low-fat high-carbohydrate diets accentuate postprandial lipemia in patients with niddm?. Diabetes Care.

[59] Wilson D.E., Chan I.F., Buchi K.N., Horton S.C. (1985). Postchallenge plasma lipoprotein retinoids: Chylomicron remnants in endogenous hypertriglyceridemia. Metabolism.

[60] Jeppesen J., Chen Y.I., Zhou M.Y., Schaaf P., Coulston A., Reaven G.M. (1995). Postprandial triglyceride and retinyl ester responses to oral fat: Effects of fructose. Am. J. Clin. Nutr..

[61] Huntriss R., Campbell M., Bedwell C. (2018). The interpretation and effect of a low-carbohydrate diet in the management of type 2 diabetes: A systematic review and meta-analysis of randomised controlled trials. Eur. J. Clin. Nutr..

[62] van der Klaauw A.A., Keogh J.M., Henning E., Trowse V.M., Dhillo W.S., Ghatei M.A., Farooqi I.S. (2013). High protein intake stimulates postprandial GLP1 and PYY release. Obesity.

[63] Woods S.C., Lutz T.A., Geary N., Langhans W. (2006). Pancreatic signals controlling food intake; insulin, glucagon and amylin. Philos. Trans. R. Soc. Lond. B Biol. Sci..

[64] Svane M.S., Jorgensen N.B., Bojsen-Moller K.N., Dirksen C., Nielsen S., Kristiansen V.B., Torang S., Wewer Albrechtsen N.J., Rehfeld J.F., Hartmann B. (2016). Peptide YY and glucagon-like peptide-1 contribute to decreased food intake after Roux-en-Y gastric bypass surgery. Int. J. Obes..

[65] Gibbons C., Finlayson G., Caudwell P., Webb D.L., Hellstrom P.M., Naslund E., Blundell J.E. (2016). Postprandial profiles of CCK after high fat and high carbohydrate meals and the relationship to satiety in humans. Peptides.

